# Pulmonary vein isolation for atrial fibrillation using true high-power short-duration vs. cryoballoon ablation

**DOI:** 10.1007/s00392-023-02188-2

**Published:** 2023-04-02

**Authors:** Jonas Wörmann, Jakob Lüker, Jan-Hendrik van den Bruck, Karlo Filipovic, Susanne Erlhöfer, Cornelia Scheurlen, Sebastian Dittrich, Jan-Hendrik Schipper, Daniel Steven, Arian Sultan

**Affiliations:** Department of Electrophysiology, Heart Center, University of Cologne, Kerpener Str. 62, 50937 Cologne, Germany

**Keywords:** High power short duration, Cryoballoon ablation, Atrial fibrillation ablation, Pulmonary vein isolation

## Abstract

**Aims:**

Pulmonary vein isolation (PVI) is achievable and effective using radiofrequency (RF) catheter (CA) or cryoballoon (CB) ablation. The newly introduced high RF-power short-duration ablation (HPSD) technique has shown promising results. Data comparing HPSD- to CB-PVI is sparse. We sought to investigate success rates and procedural differences of HPSD-PVI vs. CB-PVI in patients undergoing ablation for PAF and persAF.

**Methods:**

Consecutive patients undergoing de novo PVI (HPSD or CB) were included. A power setting of 70W/7 s (70W/5 s at posterior wall) using a flexible tip catheter with enhanced irrigation was considered as true HPSD. Follow-up consisted of out-clinic pts visits, tele-consultation, 48-h Holter ECG, app-based telemonitoring and cardiac implanted electronic devices (CIED) interrogation.

**Results:**

721 patients (46 HPSD, 675 CB) were analyzed. In all HPSD (27 persAF [59%]) and CB patients (423 persAF [63%]), PVI was successfully achieved. Procedure duration was significantly longer for HPSD (91 ± 19 min vs. 72 ± 18 min, *p* < 0.01). Ablation time was similar in both groups (HPSD: 44 ± 19 min vs. CB: 40 ± 17 min; *p* = 0.347). No major complications occurred in HPSD. For CB-PVI, in 25 (3.7%; *p* = 0.296) patients, complications occurred. At a follow-up of 290 ± 135 days, arrhythmia-free survival using HPSD was non-inferior to CB-PVI in the Kaplan–Meier survival analysis (*p* = 0.096).

**Conclusion:**

PVI using HPSD is equally effective and safe to CB-PVI. This analysis revealed a similar arrhythmia-free survival after HPSD and CB with low complication rates. Procedure duration for CB was significantly shorter while LA dwell time excluding mapping was equal. Currently, a prospective trial is conducted to corroborate these findings.

## Introduction

Pulmonary vein isolation (PVI) for the treatment of symptomatic paroxysmal and persistent atrial fibrillation (PAF and persAF) has emerged as a first-line therapy option [1]. Successful PVI is equally achieved and effective using either radiofrequency (RF) or cryoballoon (CB) catheter ablation [1, 2]. Recently, two randomized trials (STOP-AF and EARLY-AF) using CB-PVI for early invasive PAF treatment again documented the superiority of CB-PVI as opposed to medical therapy for PAF [3, 4]. Advantages of CB-PVI are shorter procedure times with similar safety and efficacy outcomes as compared to conventional RF ablation with high reproducibility due to a lower level of procedure complexity [5, 6]. Therefore, many centers prefer CB-PVI over conventional RF ablation (low energy and longer application duration) for de novo PVI [3, 4]. However, higher fluoroscopy dosage, amount of contrast medium, absent left atrial (LA) voltage display with the possibility to characterize LA substrate and the inability of treating consecutive arrhythmias beyond PVI can be considered as disadvantages of CB-PVI.

Recently, new modalities of conventional RF ablation have been introduced for PVI. The true high RF-power short-duration ablation (HPSD/70 W) technique has shown to be safe and equally effective as compared to conventional RF ablation but with significantly shorter procedure time and superior long-term outcome [7–9].

Experimental studies revealed that a power setting of 70 W with an application duration of 7 s (5 s at the posterior wall) using a flexible tip non-contact force ablation catheter with enhanced irrigation increases resistive heating with a decrease in conductive heating abilities and, therefore, generates wider and shallower tissue lesion as compared to conventional energy settings (true high power) [10]. A randomized trial comparing true HPSD with standard RF ablation of Kottmaier et al. from 2020 showed superior outcome vs. conventional RF ablation [11].

A recent meta-analysis including 10 studies with 2954 patients also revealed significantly higher rates of atrial arrhythmia-free survival (OR 1.44; *p* = 0.02) after HPSD ablation as compared to conventional RF ablation [12].

Furthermore, previous studies have reported on efficacy, safety, and outcome of conventional RF, HPSD and CB ablation to achieve PVI [11]. However, data comparing true HPSD- to CB-PVI is sparse.

We, therefore, sought to investigate success rates and procedural differences for true HPSD-PVI vs. CB-PVI in patients undergoing de novo ablation for PAF and persAF. Further, we sought to prove the hypothesis whether HPSD-PVI is as safe, fast, and effective as CB-PVI.

## Methods

### Study population

Between 01/2018 and 08/2021, all consecutive patients undergoing de-novo PVI (HPSD or CB) for symptomatic PAF or persAF were included in this retrospective analysis. Exclusion criteria were an age of < 18 years, previous left atrial ablation, and lack or withdrawal of written informed consent. Data acquisition was performed using an electronic case report form (CRF) (*RedCap Database, Nashville, Tennessee, USA*).

The trial complied with the Declaration of Helsinki, the local ethics committee approved the protocol, and all patients provided written informed consent for the procedure, general data acquisition, processing, and analysis.

### Procedure

Decision on the mode of ablation was chosen by operators’ discretion.

In both groups, oral anticoagulation was discontinued the day before ablation. In patients on vitamin K antagonists, an international normalized ratio (INR) of 2–3 was targeted. All procedures were performed under deep analgo-sedation using propofol, midazolam, and fentanyl.

After transesophageal echocardiography to rule out intracardiac thrombi, triple (HPSD group) or double (CB group) groin access was established via the right femoral vein. A decapolar catheter (Dynamic XT™, large curve 4.0/Decapolar, Boston Scientific, Marlborough, Massachusetts, USA) was positioned into the coronary sinus (CS). Transseptal puncture (TSP) was performed using *TSX*™ fixed curve transseptal sheath and TSX™ transseptal needle (Boston Scientific, Marlborough, Massachusetts, USA).

In both groups, after successful TSP, an activated clotting time (ACT) > 300 s was targeted using a weight-adjusted bolus of unfractionated heparin with repetitive boli adjusted to an every 30 min ACT measurement.

Esophageal temperature was monitored using a temperature probe (S-Cath, Esophageal Temperature Probe, Circa Scientific Inc., Englewood, Colorado, USA) in all procedures.

Ablation was stopped when esophageal-probe temperatures exceeded temperatures of > 40 °C (HPSD) or detected values < 16 °C (CB) to prevent atrio-esophageal fistula formation or esophageal erosion.

Procedure duration was defined as the time from groin puncture to sheath removal (skin-to-skin time). To receive comparable data on ablation time for complete PVI, PVI time was measured excluding three-dimensional mapping time (first burn/freeze to complete PVI). After PVI, all PV’s were checked without a waiting period in both groups.

After PVI in both groups, a figure-of-eight-suture in conjunction with compression bandage was used to prevent groin complications [13, 14].

Oral anticoagulation was continued the same day. All patients were ECG monitored for 48 h after the procedure on the electrophysiology ward.

### HPSD-PVI

All HPSD procedures were performed using a three-dimensional mapping system (Ensite Precision™ or Ensite X™, Abbott, Abbott Park, Illinois, USA). The map of the LA was acquired using a circumferential decapolar catheter (Advisor FL™ Sensor Enabled, Abbott, Abbott Park, Illinois, USA). A non-contact-force ablation catheter with enhanced tip irrigation (20 ml/min) and distally positioned thermocouples (Flexability D- or F-Curve; Abbott; Abbott Park, Illinois, USA) was used due to its favorable design for true HPSD ablation and thermal tissue conduction and measurement [7, 15]. Antral PVI was achieved using point-by-point ablation with isolation of both PV pairs avoiding overlap of the ablation lesion projections. A power setting of 70 W for 7 s was used at all LA sites except for the posterior wall (duration reduced to 5 s) [7, 10].

Circumferential PVI (entrance block) was monitored with the circumferential mapping catheter. The endpoint was defined as complete PVI (entrance and if applicable exit block) with the additional endpoint of unexcitability of the ablation line evaluated by pacing along the ablation line [16].

### CB-PVI

All CB procedures were performed using the FlexCath Advance™ sheath and the ArcticFront Advance Pro™ CB (Medtronic; Minneapolis, Minnesota, USA). PV occlusion was evaluated fluoroscopically using contrast medium application via the CB catheter. To obtain a homogenous comparator cohort, all CB-PVI were performed using the third generation CB. Assessment of electrical isolation was performed via a decapolar circumferential mapping catheter positioned in the PV antrum (Achieve Advance™, Medtronic; Minneapolis, MN, USA). The freeze duration was determined either by time to isolation (TTI) or by the nadir temperature during freeze application [17, 18].

During freezes of the right PVs, phrenic nerve pacing using a CS catheter was performed with diaphragmatic compound motor action potential monitoring (CMAP) to avoid phrenic nerve palsies [19]. The endpoint of CB-PVI was entrance block of all PVs revealed by the mapping catheter.

### Follow-up

Follow-up consisted of out-clinic patients’ visits 3 and 12 months after PVI, photoplethysmogram (PPG) app-based tele-consultation (Fibricheck™, Hasselt, Belgium) [20], 48-h Holter ECG and CIED interrogation if applicable. Any detected atrial arrhythmia (atrial fibrillation, atrial tachycardia, atrial flutter) longer than 30 s was defined as recurrence of arrhythmia after a 90-day blanking period.

### Endpoints

The primary endpoint was defined as arrhythmia-free survival during follow-up. Secondary endpoints were procedural differences (procedure duration, ablation time, fluoroscopy-time and -dose, use of contrast medium) and complications.

### Statistics

Statistical analysis was performed using SPSS for Mac version 28.0 and Excel for Mac version 16.57. Continuous variables are presented as mean ± standard deviation.

Categorical variables as percentages. Univariate analysis was performed using *t* test and Chi-squared test. Multivariate analysis was performed using Kaplan–Meier survival and cox-regression.

A *p* value < 0.05 was considered statistically significant.

## Results

### Study population

A total of 721 patients (46 HPSD, 675 CB) were analyzed. Baseline characteristics are displayed in Table 1. The average age was 62 ± 4 years in HPSD and 66 ± 13 years in CB patients (*p* = 0.627). In the HPSD group, 17 patients were female (37%) vs. 264 (39%) in the CB group (*p* = 0.876).

**Table 1:** Baseline data of HPSD and CB patients

	HPSD (*n* = 46)	CB (*n* = 675)	*p* value
Age (year)	62 ± 4	66 ± 13	0.627
Women [%]	17 [37%]	264 [39%]	0.876
PAF [%]	19 [41%]	252 [37%]	0.638
LV-EF (%)	55.6 ± 6.4	56.4 ± 8.5	0.531
CHADS-VASc	2	2	1.000
LA diameter (mm)	39.0 ± 5.7	39.5 ± 6.3	0.955
CAD [%]	8 [17.4%]	137 [20.3%]	0.708
HTN [%]	30 [65.2%]	458 [67.9%]	0.745
DM [%]	5 [10.9%]	79 [11.7%]	1.000
GFR (ml/min)	77 ± 20	77 ± 65	1.000
AADs [%]	10 [21.7%]	213 [31.6%]	0.189
Amiodarone [%]	8 [17.4%]	172 [25.5%]	0.290
Flecainide [%]	2 [4.3%]	41 [6.1%]	1.000

*CAD* Coronary artery disease, *HTN* hypertension, *DM* diabetes mellitus, *AAD* antiarrhythmic drug

There was no statistically significant differences in the distribution of PAF and persAF patients (HPSD: 19 PAF [41%], 27 persAF [59%]; CB: 252 PAF [37%], 423 persAF [63%]; *p* = 0.638).

Baseline data showed no significant differences in co-morbidities and patient characteristics (Table 1).

At the time of procedure, 10 patients [21.7%] in the HPSD group were on AADs (8 amiodarone, 2 flecainide) as compared to 213 [31.6%] (172 amiodarone, 41 flecainide) in the CB group showing no significant difference between groups (*p* = 0.189). Left atrial (LA) diameter (HPSD: 39.0 ± 5.7 mm vs. CB: 39.5 ± 6.3 mm; *p* = 0.955) and left ventricular ejection fraction (LV-EF) (HPSD: 55.6 ± 6.4% vs. CB 56.4 ± 8.5; *p* = 0.531) were comparable.

### Outcome

**Fig. 1:**
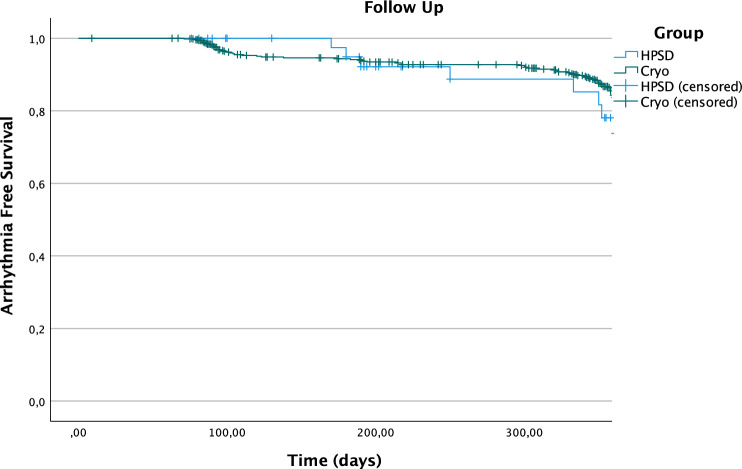
Kaplan–Meier analysis showing arrhythmia-free survival during follow-up in true HPSD and CB patients; Chi-squared 2.777 (*p* = 0.096)

After a mean follow-up of 290 ± 135 days, the Kaplan–Meier analysis (displayed in Fig. 1) showed no significant differences in arrhythmia-free survival between both groups. The log-rank analysis showed a Chi-squared of 2.777 (*p* = 0.096) indicating no significance in the multivariate analysis. The distribution of patients receiving AAD’s during follow-up was comparable in both groups (HPSD 14 [30.4%] vs. CB 213 [31.6%]; *p* = 1.000) (Table 2).

**Table 2:** Outcome data of true HPSD and CB patients

	HPSD (*n* = 46)	CB (*n* = 675)	*p* value
Free from arrhythmia [%]	38 [82.6%]	458 [67.9%]	0.047
AAD’s during FU	14 [30.4%]	213 [31.6%]	1.000

Mean follow-up was 290 ± 135 days

*AAD* Antiarrhythmic drug

In the descriptive analysis (Table 2), significantly more patients were free from any atrial arrhythmia after a single procedure undergoing HPSD-PVI (38 HPSD [82.6%] vs. 458 after CB-PVI [67.9%]; *p* = 0.047).

### Procedural data

**Fig. 2:**
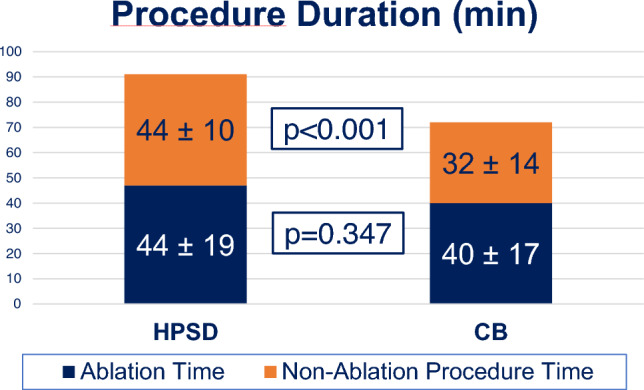
Procedure duration in true HPSD and CB. Ablation time was equal in both groups

In both groups (HPSD and CB), PVI was successfully achieved in all patients (100%). Procedural and safety outcome data are displayed in Table 3. Procedure duration was significantly longer in HPSD patients (91 ± 19 min vs. 72 ± 18 min, *p* < 0.001) as compared to CB. Ablation time displayed in Fig. 2 was 44 ± 19 min for HPSD and 40 ± 17 min for CB without significant difference (*p* = 0.347). Fluoroscopy time (HPSD 14 ± 5 min and CB 14 ± 7 min; *p* = 1) and dose (HPSD 3798 ± 2460 mGy cm^2^; CB 3199 ± 4138 mGy cm^2^; *p* = 0.333) was comparable in both groups. The amount of contrast medium was significantly lower in HPSD procedures (16.8 ± 8.1 ml vs. CB: 53.9 ± 32.8 ml; *p* < 0.001).

**Table 3:** Procedural data of true HPSD and CB patients

	HPSD (*n* = 46)	CB (*n* = 675)	*p* value
Duration (min)	91 ± 19	72 ± 18	< 0.001
LA dwell time (min)	44 + 19	40 ± 17	0.347
Fluoroscopy (min)	14 ± 5	14 ± 7	1.000
Dose (mGy cm^2^)	3798 ± 2460	3199 ± 4138	0.333
Contrast medium (ml)	16.8 ± 8.1	53.9 ± 32.8	< 0.001
Complications [%]	0 [0%]	25 [3.7%]	0.296
Bleedings [%]	0 [0%]	16 [2.4%]	0.616
Phrenic palsy [%]	0 [0%]	7 [1.0%]	1.000
Tamponade [%]	0 [0%]	2 [0.3%]	1.000
Death [%]	0 [0%]	1 [0.15%]	1.000

Procedure duration was significantly shorter in CB patients. LA dwell time excludes mapping time. Phrenic palsies were all transient

For HPSD, a mean of 111 ± 42 RF ablations were performed (RF duration: 784 ± 452 s; total RF energy: 39,335 ± 20,171 W). For CB procedures, PVI was achieved using a median of 5 (5–7) freezes. Total freeze time was 264 ± 158 s.

No major complications were reported for the HPSD group. In the CB group in 25 (3.7%) patients, complications occurred, 16 (2.4%) groin bleedings, 7 (1.0%) transient phrenic nerve palsies, and 2 (0.3%) cardiac tamponades of which 1 was lethal later during the clinical course after the attempt of surgical correction. The complication occurred after TSP and was presumably unrelated to the ablation device (*p* = 0.296).

## Discussion

### Main findings

This study reveals that true HPSD ablation for PVI using 70 W for 5–7 s is equally safe, effective, and efficient compared to CB-PVI in both, PAF and persAF patients.

To our knowledge, this analysis provides for the first time the data comparing true HPSD (70 W) to CB-PVI in a typical AF population.

The arrhythmia-free survival for true HPSD-PVI after 1 year was non-inferior compared to CB-PVI in PAF and persAF patients. These findings are in line with a randomized trial comparing true HPSD with standard RF-ablation by Kottmaier et al. with a similar arrhythmia-free survival of 83.1% (compared to 82.6% in our analysis) showing a superior outcome vs. conventional RF ablation [11].

Regarding secondary endpoints, the procedure duration was significantly longer in our study in the HPSD group than in CB ablation while fluoroscopy time and dose were similar.

Contrary to our data, prior trials showed shorter radiation times for RF and HPSD ablation [11]. This might be explained by the fact that we included our first HPSD procedures in our analysis. The longer radiation times might arise from the learning curve with the non-contact-force flexible tip catheter.

The longer procedure time for true HPSD procedures was driven by the 3D-LA-mapping time. Hence, ablation time did not show any statistical difference between both groups. This indicates that true HPSD is as fast as CB comparing the mere time from first burn or freeze, respectively, to complete PVI. In addition, true HPSD enables to target concomitant (e.g., atrial flutter) or consecutive arrhythmias after PVI within the first procedure, foremost in patients with persAF undergoing catheter ablation.

The lower contrast medium but comparable outcome and procedure time favors true HPSD over CB in patients suffering from severe chronic kidney disease.

Up to date, only one single-center randomized controlled trial by Pak et al. compared “moderate” HPSD (50–60 W) to CB showing similar outcomes in AF recurrences after 1 year and no differences in procedure safety. Of note, the authors reported a significantly longer procedure time for HPSD compared to CB-PVI. Two factors might explain the longer procedure time as reported: First, in 98.1% of the HPSD patients, an additional CTI ablation was performed further prolonging the procedure duration vs. no CTI in the CB group [21].

Second, the “moderate” power settings used in the Pak et al. trial (50–60 W) do not correspond to our definition of true HPSD (70 W/7 s) resulting in a prolonged lesion creation and, therefore, longer procedure duration. An analysis of the net LA dwell time might also reveal no difference for both groups.

Lesion metrics obtained by true HPSD with enhanced tip irrigation catheters are broader, shallower, and more homogenic compared to standard settings leading to a smaller number of PV gaps [10, 11].

In a trial performed by Kurose et al., the number of visual gaps shown in Late-Gadolinium-Enhancement (LGE) MRI after CB was even higher than after conventional RF ablation which might indicate possible advantages in outcome parameters of true HPSD compared to CB-PVI [22]. This broad and shallow lesion formation created by true HPSD may partly explain the non-inferiority in outcome compared to CB-PVI.

In our analysis, both groups showed very few procedural complications. In the HPSD group, no complication occurred. The reported groin complications in the CB might be explicable by the larger sheath size.

The rate of phrenic nerve palsy was slightly lower in our analysis compared to a large multicenter registry with 17,356 patients that showed an incidence of 4.2%. The complete recovery is in line with the registry data (97% recovery after 1 year) [23].

Of the tamponades in the CB group, one was managed by pericardiocentesis and one died due to severe preexisting conditions and the course of this complication requiring surgical intervention. To reveal any possible differences between both techniques, larger data will have to be analyzed.

### Limitations

These data are of retrospective nature and do not provide a randomized controlled comparison of both techniques. Therefore, a randomized controlled trial comparing true HPSD to CB in PAF patients is ongoing in our center (HIPAF-trial) and will provide missing prospective data (ClinicalTrials.gov: NCT04855890) on this topic. Though baseline data of both groups were comparable, it would be desirable to evaluate groups with similar sizes, i.e., a larger HPSD population. Not many centers perform true HPSD with 70 W/7 s; therefore, to obtain multicenter data for analysis will remain a challenge [12].

Since cerebral imaging for silent cerebral ischemia is not part of the clinical practice on our center, we cannot provide data for silent brain ischemia. To our knowledge, there is no existing data for silent cerebral ischemia in HPSD so far, while incidence in CB and conventional RF ranges from 4.8 to 38.4% with no significant differences between both technologies, and thus far no proven correlation to relevant clinical outcome measures [24].

## Conclusion

PVI using true HPSD is equally effective, safe, and efficient compared to CB-PVI in patients with PAF and persAF using less contrast medium than CB. This analysis revealed a similar arrhythmia-free survival after true HPSD as compared to CB-PVI with low complication rates in this relatively small true HPSD cohort. The overall procedure duration for HPSD was significantly longer compared to CB while ablation time did not reveal any significant difference. Currently, a prospective trial is conducted to corroborate these findings.

## References

[1] Hindricks G, Potpara T, Dagres N, Arbelo E, Bax JJ, Blomström-Lundqvist C (2021). 2020 ESC Guidelines for the diagnosis and management of atrial fibrillation developed in collaboration with the European Association for Cardio-Thoracic Surgery (EACTS)The Task Force for the diagnosis and management of atrial fibrillation of the European Society of Cardiology (ESC) Developed with the special contribution of the European Heart Rhythm Association (EHRA) of the ESC. Eur Heart J.

[2] Kuck K-H, Brugada J, Fürnkranz A, Metzner A, Ouyang F, Chun KRJ (2016). Cryoballoon or radiofrequency ablation for paroxysmal atrial fibrillation. N Engl J Med.

[3] Wazni OM, Dandamudi G, Sood N, Hoyt R, Tyler J, Durrani S (2021). Cryoballoon ablation as initial therapy for atrial fibrillation. N Engl J Med.

[4] Andrade JG, Wells GA, Deyell MW, Bennett M, Essebag V, Champagne J (2021). Cryoablation or drug therapy for initial treatment of atrial fibrillation. N Engl J Med.

[5] Maurer T, Schlüter M, Kuck KH (2020). Keeping it simple: balloon devices for atrial fibrillation ablation therapy. JACC Clin Electrophysiol.

[6] Providencia R, Defaye P, Lambiase PD, Pavin D, Cebron JP, Halimi F (2017). Results from a multicentre comparison of cryoballoon vs. radiofrequency ablation for paroxysmal atrial fibrillation: is cryoablation more reproducible?. Europace.

[7] Kottmaier M, Popa M, Bourier F, Reents T, Cifuentes J, Semmler V (2019). Safety and outcome of very high-power short-duration ablation using 70 W for pulmonary vein isolation in patients with paroxysmal atrial fibrillation. Europace.

[8] Leshem E, Zilberman I, Tschabrunn CM, Barkagan M, Contreras-Valdes FM, Govari A (2018). High-power and short-duration ablation for pulmonary vein isolation: biophysical characterization. JACC Clin Electrophysiol.

[9] Reddy VY, Grimaldi M, De Potter T, Vijgen JM, Bulava A, Duytschaever MF (2019). Pulmonary vein isolation with very high power, short duration, temperature-controlled lesions: the QDOT-FAST trial. JACC Clin Electrophysiol.

[10] Bourier F, Duchateau J, Vlachos K, Lam A, Martin CA, Takigawa M (2018). High-power short-duration versus standard radiofrequency ablation: insights on lesion metrics. J Cardiovasc Electrophysiol.

[11] Kottmaier M, Popa M, Bourier F, Reents T, Cifuentes J, Semmler V (2020). Safety and outcome of very high-power short-duration ablation using 70 W for pulmonary vein isolation in patients with paroxysmal atrial fibrillation. EP Eur.

[12] Ravi V, Poudyal A, Abid QUA, Larsen T, Krishnan K, Sharma PS (2021). High-power short duration vs conventional radiofrequency ablation of atrial fibrillation: a systematic review and meta-analysis. EP Eur..

[13] Aytemir K, Canpolat U, Yorgun H, Evranos B, Kaya EB, Sahiner ML (2016). Usefulness of ‘figure-of-eight’ suture to achieve haemostasis after removal of 15-French calibre femoral venous sheath in patients undergoing cryoablation. EP Eur.

[14] Jensen CJ, Schnur M, Lask S, Attanasio P, Gotzmann M, Kara K (2020). Feasibility of the figure-of-8-suture as venous closure in interventional electrophysiology: one strategy for all?. Int J Med Sci.

[15] Winterfield JR, Jensen J, Gilbert T, Marchlinski F, Natale A, Packer D (2016). Lesion size and safety comparison between the novel flex tip on the FlexAbility ablation catheter and the solid tips on the ThermoCool and ThermoCool SF ablation catheters. J Cardiovasc Electrophysiol.

[16] Steven D, Sultan A, Reddy V, Luker J, Altenburg M, Hoffmann B (2013). Benefit of pulmonary vein isolation guided by loss of pace capture on the ablation line: results from a prospective 2-center randomized trial. J Am Coll Cardiol.

[17] Aryana A, Mugnai G, Singh SM, Pujara DK, De Asmundis C, Singh SK (2016). Procedural and biophysical indicators of durable pulmonary vein isolation during cryoballoon ablation of atrial fibrillation. Hear Rhythm.

[18] Ciconte G, Mugnai G, Sieira J, Velagic V, Saitoh Y, Irfan G (2015). On the quest for the best freeze: predictors of late pulmonary vein reconnections after second-generation cryoballoon ablation. Circ Arrhythm Electrophysiol.

[19] Franceschi F, Koutbi L, Mancini J, Attarian S, Prevôt S, Deharo JC (2013). Novel electromyographic monitoring technique for prevention of right phrenic nerve palsy during cryoballoon ablation. Circ Arrhythm Electrophysiol.

[20] Gruwez H, Evens S, Proesmans T, Smeets C, Haemers P, Pison L et al (2021) Head-to-head comparison of proprietary PPG and single-lead ECG algorithms for atrial fibrillation detection. EP Eur 23(Supplement_3)

[21] Pak HN, Park JW, Yang SY, Kim TH, Uhm JS, Joung B (2021). Cryoballoon versus high-power, short-duration radiofrequency ablation for pulmonary vein isolation in patients with paroxysmal atrial fibrillation: a single-center, prospective, randomized study. Circ Arrhythmia Electrophysiol..

[22] Kurose J, Kiuchi K, Fukuzawa K, Takami M, Mori S, Suehiro H (2020). Lesion characteristics between cryoballoon ablation and radiofrequency ablation with a contact force-sensing catheter: late-gadolinium enhancement magnetic resonance imaging assessment. J Cardiovasc Electrophysiol.

[23] Heeger CH, Sohns C, Pott A, Metzner A, Inaba O, Straube F (2022). Phrenic nerve injury during cryoballoon-based pulmonary vein isolation: results of the worldwide YETI registry. Circ Arrhythm Electrophysiol.

[24] Nakamura T, Okishige K, Kanazawa T, Yamashita M, Kawaguchi N, Kato N (2017). Incidence of silent cerebral infarctions after catheter ablation of atrial fibrillation utilizing the second-generation cryoballoon. EP Eur.

